# iW-Net: an automatic and minimalistic interactive lung nodule segmentation deep network

**DOI:** 10.1038/s41598-019-48004-8

**Published:** 2019-08-12

**Authors:** Guilherme Aresta, Colin Jacobs, Teresa Araújo, António Cunha, Isabel Ramos, Bram van Ginneken, Aurélio Campilho

**Affiliations:** 1 INESC TEC - Institute for Systems and Computer Engineering, Technology and Science, Rua Doutor Roberto Frias, 4200-465 Porto, Portugal; 2 Faculty of Engineering of University of Porto, Rua Doutor Roberto Frias, 4200-465 Porto, Portugal; 3 Radboud University Medical Center, 6525 Nijmegen, The Netherlands; 4 University of Trás-os-Montes e Alto Douro, Quinta de Prados, 5001-801 Vila Real, Portugal; 5 Faculty of Medicine of University of Porto, Alameda Prof. Hernâni Monteiro, 4200-319 Porto, Portugal

**Keywords:** Computed tomography, Biomedical engineering, Computer science

## Abstract

We propose iW-Net, a deep learning model that allows for both automatic and interactive segmentation of lung nodules in computed tomography images. iW-Net is composed of two blocks: the first one provides an automatic segmentation and the second one allows to correct it by analyzing 2 points introduced by the user in the nodule’s boundary. For this purpose, a physics inspired weight map that takes the user input into account is proposed, which is used both as a feature map and in the system’s loss function. Our approach is extensively evaluated on the public LIDC-IDRI dataset, where we achieve a state-of-the-art performance of 0.55 intersection over union vs the 0.59 inter-observer agreement. Also, we show that iW-Net allows to correct the segmentation of small nodules, essential for proper patient referral decision, as well as improve the segmentation of the challenging non-solid nodules and thus may be an important tool for increasing the early diagnosis of lung cancer.

## Introduction

Lung cancer is the most fatal cancer type in both men and women^1^. Thankfully, early diagnosis of this pathology and proper medical follow-up allow to increase the patients’ survival rate. Namely, annual screening of risk groups with low-dose chest computed tomography (LDCT) allows to reduce lung cancer mortality by 20%^2^. During screening, radiologists search for lung nodules by visually inspecting the LDCT volumes. Potential findings are then characterized in terms of dimension (axes length and volume), texture (solid, sub-solid and non-solid), spiculation, calcification and location. Patient follow-up is then decided according to a specific lung cancer screening guideline. Particularly, the initial nodule dimensions and growth-rate are two pivotal characteristics in major screening guidelines^3–5^ and thus accurate 3D lung nodule segmentation is an important task during screening. However, performing accurate manual segmentation is a highly time consuming task, thus motivating the need for automatic lung nodule segmentation solutions. Furthermore, it is known that nodule segmentation is a subjective task and specialists often disagree on their annotations^6^. Consequently, interactive segmentation tools are of high interest on this clinical setting. Over the past years, several automatic lung nodule segmentation methods have been proposed with the goal of automating lung cancer screening. Despite achieving acceptable performances, lung nodule segmentation methods are still limited because either do not allow for user interaction, are slow or require extensive user interaction (*e.g*. adjustment of several parameters) to achieve a satisfying result.

Segmentation methods usually take advantage of the natural characteristics of solid nodules, which commonly have high contrast with the lung parenchyma and spherical shapes. A common approach is to do voxel-wise segmentation by extracting intensity^7,8^ and shape-related features, namely from Hessian matrices^9^, and training classifiers such as Support Vector Machines or Neural Networks^10^ to obtain the final result. However, the extension of feature-design approaches for non-solid and sub-solid nodules is a hard and tedious process^11^ due to the cloudy texture, irregular shape and reduced contrast with the parenchyma of non-solid, and the diffused boundaries of sub-solid nodules.

Because of this, Convolutional Neural Networks (CNNs) have become the standard approach for medical image segmentation since they allow to significantly reduce the required field-knowledge and thus the need for manual feature design. For instance, Wang *et al*.^12^ proposed a multi-scale CNN that performs voxel-wise predictions, inside a cube containing a lung nodule, of the abnormal tissue. Each predicted voxel corresponds to the center of a fixed size patch to be processed by the network and thus predicting an entire segmentation requires the evaluation of a high number of patches. Furthermore, this model has an inherent lack of global context, since the network only evaluates patches, and thus the 3D reconstruction of the nodule may be affected. A common solution is to adapt 3D U-Net^13^ architectures, since they allow to consider both local and global context. With this in mind, Wu *et al*.^14^ proposed a multi-task scheme for pulmonary nodule segmentation together with the prediction of the nodules’ expected malignancy, achieving state-of-the-art performance in both tasks. This malignancy prediction is performed by concatenating and processing, via a set of fully-connected layers, the features of the segmentation network’s bottle neck with a convolved version of the produced segmentation prediction.

Despite the high performance of deep learning methods, their application in the medical field is being criticized due to (1) the inherent lack of explanations behind the decision and, (2) the production of deterministic outputs, ignoring the existing inter-observer variability of the annotations and inhibiting the medical specialist to interact and change the decisions of the system. With this in mind, Kohl *et al*.^15^ proposed to model the inter-observer variability by combining a conditional variational auto encoder (cVAE) with an U-Net. The cVAE is used for drawing a set of feature maps sampled from the trained latent space representation. These features are then concatenated with the last feature maps of the U-Net, which are then convolved to produce the segmentation output. By varying the sampled set of features from the cVAE, this model is capable of producing different, yet plausible, nodule segmentations. However, the method of Kohl *et al*. does not allow the clinician to alter the segmentation, instead forcing the specialist to opt for the result closer to his/her expectations.

Recently, Wang *et al*.^16^ proposed a scribble-based approach to refine 2D and 3D segmentations resulting from a fully-convolutional neural network. First, the user selects a bounding box containing the anatomical structure to segment. For each unseen image, the top of a pre-trained segmentation model is trained to accommodate the foreground and background scribbles by minimizing, via an expectation-maximization (EM) approach, a loss function composed of two terms: (1) a pixel-wise weighted categorical cross-entropy term that prioritizes the inclusion of foreground and the removal of background scribbles, and (2) a pair-wise smoothness term that encourages the aggregation of neighbor pixels of similar intensity^17^. Even though this scheme achieves state-of-the-art results on organ segmentation in MRI images, its application for lung nodule segmentation is limited due to the nature of the abnormalities. For instance, nodules are often attached to structures of similar intensity, such as the pleural wall and blood vessels, and thus the EM scheme may lead to the inclusion of these structures in the segmentation and thus potentially demand extra manual correction efforts. Also, sub-solid and non-solid nodules do not have a clear boundary, which can further hinder the minimization of the smoothness term.

**Figure 1:**
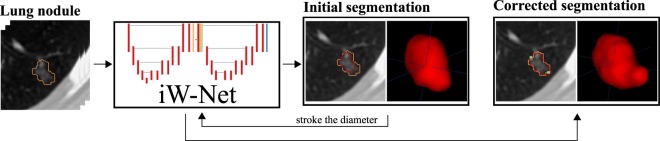
Automatic and interactive lung nodule segmentations using iW-Net.  ground-truth;  prediction;  end-points.

With this in mind, we propose an end-to-end deep learning scheme, iW-Net (interactive W-Net), that allows for both automatic and optional interactive 3D lung nodule segmentation, as suggested in Fig. 1. The network receives as input a cube of fixed dimensions which centroid is indicated by the user, or by an automatic nodule detection framework, and proposes a corresponding segmentation. If the user is not satisfied, the segmentation can be corrected by using the end-points of a manually inserted stroke of the nodule’s diameter. For this purpose, we use a second segmentation network that integrates the 3D image of the nodule, the initial segmentation and the coordinates of the end-points. Namely, this paper shows that the end-points can be represented by a physics-inspired weight map ${\mathcal M}$ that, when used as a feature map and as loss function term, allows to cap the inter-observer agreement in the LIDC-IDRI public dataset. Our approach allows a simple and fast segmentation correction when that information is available without introducing a significant over-head in comparison to the non-guided version of the model.

## Results and Discussion

### Experiment 1 - comparison with 3D U-Net

iW-Net without user interaction outperforms the baseline 3D U-Net^13^. As shown in Table 1, the nodule segmentation performance is relatively increased by approximately 26% while reducing the number of parameters by a factor of 12. In fact, the reduction of the size of the network contributed to the disparity between the referred IoUs by allowing to increase the batch size during training. The larger batch size allows for a more robust batch normalization, easing the error’s back-propagation and thus improving the convergence and performance of the iW-Net in comparison to 3D U-Net.

**Table 1:** Intersection over Union ± the standard deviation of the prediction of the first block iW-Net in comparison to a 3D U-Net and the inter-observer agreement.

	IoU	Number of parameters
Inter-observer	0.59 ± 0.14	—
3D U-Net^13^	0.38 ± 0.08	19 080 001
iW-Net first block	0.48 ± 0.19	1 592 093

**Figure 2:**
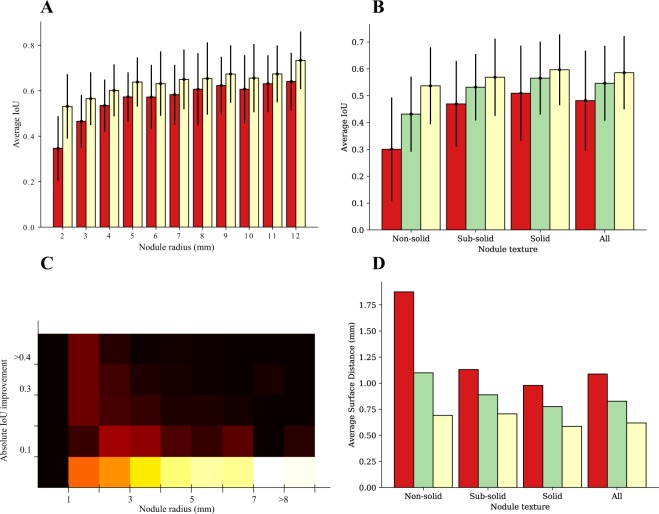
(**A**) Average Intersection over Union per nodule radius for the initial segmentation of iW-Net () and the inter-observer agreement (), and the respective standard deviation; (**B**) Average Intersection over Union per nodule texture for iW-Net’s initial () and corrected segmentations (), the inter-observer agreement (), and the respective standard deviation; (**C**) Average absolute Intersection over Union improvement between the initial and the corrected segmentation using iW-Net per nodule radius. Each column is normalized according to the respective number of nodules. Colorbar: 0  1; (**D**) Average surface distance (ASD) per nodule texture using iW-Net for the initial segmentation (), corrected segmentation () and the inter-observer agreement ().

As expected, iW-Net’s prediction without user-interaction tends to be better for larger nodules (see Fig. 2A). Indeed, since most segmentation errors occur near the nodules’ boundary, then smaller nodules, which have a higher surface area *vs* volume ratio, should be more challenging. Interestingly, the inter-observer agreement follows the same tendency, indicating that smaller nodules are particularly difficult to segment.

### Experiment 2 - user interaction assessment

**Figure 3:**
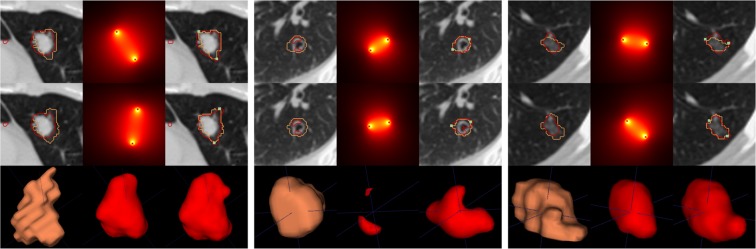
Examples of segmentations proposed by iW-Net. For each of the 3 × 3  block:  ground-truth () and output of the first block of iW-Net () for two different annotators;  weight maps ${\mathcal M}$ based on the end-points of the diameter;  resulting segmentations after considering the diameter’s end-points ();  example of a 3D representation of the ground-truth from the nodule above;  3D representation of the initial segmentation;  3D representation of the guided segmentation.

The proposed simplistic user interaction approach allows to improve the baseline segmentation on more than 75% of the cases. Figure 3 depicts examples where iW-Net allows to significantly alter the 3D shape of the segmentation just by the introduction of two points, being capable of correcting, at least partially, poor segmentations (middle) as well as change the orientation of the proposed region of interest (right). In fact, 44% of the user-introduced points are inside the new segmentations, further showing the tendency of iW-Net to alter the shape of the segmentation. Also, as detailed in Table 2 and Fig. 2B, iW-Net specially enables the delineation correction of the challenging non-solid nodules.

**Table 2:** Percentage of the number of improved segmentations and respective Average absolute intersection over union increase (IoU improv) ± the standard deviation of iW-Net’s guided segmentation in comparison to the initial segmentation.

Nodule type	All	Solid	Sub-solid	Non-solid
Improv. (%)	78	78	73	87
IoU improv.	0.08 ± 0.10	0.07 ± 0.08	0.08 ± 0.09	0.15 ± 0.13

Our proposed approach also has promising results for computer-aided lung cancer screening. As depicted in Fig. 2C, the radius range [1, 4](*mm*) is where iW-Net (user supervised) most improves the quality of the nodules’ segmentation. Importantly, several international lung cancer screening guidelines, such as LUNG-RADS^3^, point this dimension range as essential to classify a nodule as either benign or malignant.

iW-Net with the simulated user-interaction allows to improve over the baseline for nodules of different dimensions and textures, as summarized in Figs 2B,C and 3. However, the achieved IoU is still, in average, 0.04 lower than the inter-observer agreement. A possible reason for this is that, due to the variability of the ground-truth in the data (*i.e*. several segmentations for the same nodule), the network is likely to learn an average segmentation in order to minimize the loss over the redundant training images. Also, during the segmentation correction we are always selecting the two furtherest points in the nodule boundary. In fact, this is a challenging scenario since there is no guarantee that the selected points are in the direction in which the segmentation needs to be corrected. Instead, we are assuming that providing an estimation of the nodule’s largest axis is sufficient to improve the segmentation.

Despite always using the two farthest points to correct the segmentation, iW-Net improves the baseline segmentation’s ASD for all nodule types by 24%, (Fig. 2D). Namely, the baseline’s average ASD is 1.09 and the corrected’s is 0.827, meaning that iW-Net has a segmentation error that is in average less than 1 voxel. Also, similarly to the IoU’s behavior, the simplistic user interaction allows to significantly improve the quality of the nodules’ segmentation in non-solid and sub-solid abnormalities.

### Comparison with other approaches

iW-Net achieves a performance in pair to the inter-observer agreement, similarly to other state-of-the-art approaches. Note that making a direct comparison between the approaches is non-trivial since (1) there is a great variation on the size of the test set, type and size of the nodules used as well as the minimum inter-observer agreement; (2) different methods use different voxel scales, and the inherent re-sampling affects the shape of the ground-truth; (3) there are different ways of combining the ground-truth annotations from the different observers (using all, the average or the median, for instance) to produce the final evaluation mask. Nevertheless, for reference, Table 3 shows the achieved IoUs of different approaches on the LIDC-IDRI dataset. Similarly to other state-of-the-art approaches, the performance of our method is close to the inter-observer agreement, even though a significantly larger number of samples has been studied. Advantageously, iW-Net does not rely on computationally heavy pre-processing steps and allows to segment nodules of all sizes and textures without the need to define bounding boxes or other specific parameters. In fact, the average inference time per nodule is only 0.12 ± 0.08 (s). Furthermore, our system allows to correct segmentations without requiring an external algorithm, which none of the others do. Finally, unlike Wu *et al*.^14^ model, training iW-Net does not require other metadata, making it easier to enrich the training set and thus the generalization capability of the system.

**Table 3:** Average Intersection over Union ± the standard deviation for lung nodule segmentation methods on the LIDC-IDRI dataset, and the reported inter-observer agreement (Inter).

Approach	Year	# Nodules		IoU	Inter
		Train	Test		
Tan *et al*.^8^	2013	NA	23	0.65	NA
Lassen *et al*.^11^*	2015	NA	19	0.52 ± 0.07	0.54 ± 0.05
Messay *et al*.^10^	2015	300	66	0.74 ± 0.11	NA
Gonçalves *et al*.^9^	2016	57	512	0.71 ± 0.07	0.71 ± 0.1
Wang *et al*.^12^	2017	350	493	0.71 ± 0.12	0.72 ± 0.04
Wu *et al*.^14^	2018	1404	1404	0.58 ± 0.02	NA
iW-Net	2018	1593	1593	0.55 ± 0.14	0.59 ± 0.14

NA: information is not available. *Sub-solid nodules only.

### Hyper-parameters

**Figure 4:**
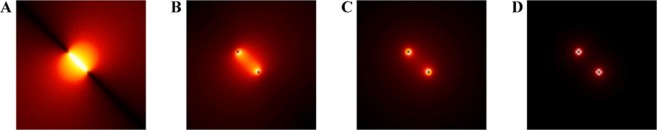
Examples of weight maps (middle slice is shown) with different decay values *p*. (**A**) *p* = 0; (**B**) *p* = 0.5; (**C**) *p* = 1; (**D**) *p* = 2; Colorbar: 0  1.

The best performing set of parameters, selected by random search, are *γ* = 0.59, *p* = 0.44 and *λ*_1_ = 0.68. These allow to achieve an average validation IoU of 0.59 in the first train/test split. Intuitively, a *p* near 0.5 (see Fig. 4B) allows to create a weight map that prioritizes the inclusion of the points and the respective connection region without overspreading (Fig. 4A) or over-emphasizing the points (Fig. 4C,D). Likewise, the found *γ* allows the binarized weight map to have an ellipsoidal structure, following the approximate shape of most of the nodules. Finally, *λ*_1_ balances the contribution of the initial manual segmentation and the added weight map during model training. In the limit where *λ*_1_ = 0 the network would be trying to approximate the nodule segmentation to an ellipsoid. On the other hand, *λ*_1_ = 0.68 ensures that the manual segmentation is the prioritized target during training and that the weight map ${\mathcal M}$ (see Fig. 3) is used for local corrections.

## Methods

**Figure 5:**
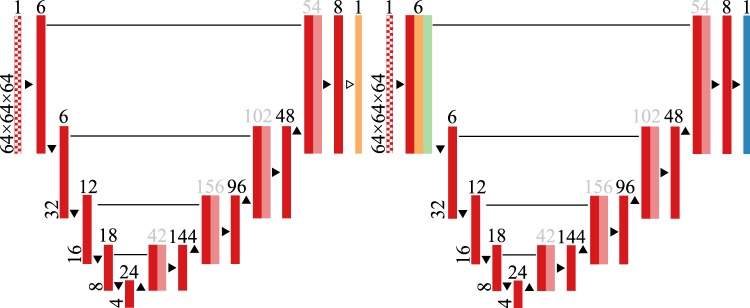
iW-Net: a network for guided segmentation of lung nodules, composed by a block responsible for predicting the initial segmentation and a second block for its correction. *S* is the side of the feature map.  input image  intermediary feature maps;  initial segmentation prediction;  weight map ${\mathcal M}$ computed from the user’s input;  corrected segmentation. ▸ 3 × 3 × 3 × *N* convolution, followed by batch normalization and rectified linear unit activation (*N* is the number of feature maps, indicated on the top of each layer); ▾ 3 × 3 × 3 × *N* convolution with stride 2 × 2 × 2, followed by batch normalization and rectified linear unit activation; ▴ 2 × 2 × 2 nearest neighbor up-sample; ▻ 3 × 3 × 3 × *N* convolution with sigmoid activation.

iW-Net allows to easily correct lung nodule segmentations according to the specialists’ perception. As depicted in Fig. 5, iW-Net first performs an (1) automatic 3D segmentation of lung nodules, predicted by the first block (*i.e*. U) of the network, and after an (2) optional segmentation correction, which is performed by the second block of the model by processing the end-points of a manually drawn nodule diameter. For this, we propose a pixel-wise weight map ${\mathcal M}$ to guide the segmentation, as detailed in Section Weight map for segmentation control. ${\mathcal M}$ is then used as a feature map of iW-Net and in a loss function term to train an auto-encoder segmentation network, as described in Sections iW-Net for nodule segmentation and Loss function.

### Weight map for segmentation control

Our weight map ${\mathcal M}$ is inspired on the attraction field generated by punctual electric charges of opposite value. Let *S* define a sphere of undetermined radius:

1 $$S(x,y,z)={(x-{x}_{0})}^{2}+{(y-{y}_{0})}^{2}+{(z-{z}_{0})}^{2}$$

where (*x*_0_, *y*_0_, *z*_0_) is the center of the sphere and (*x*_*i*_, *y*_*i*_, *z*_*i*_) are Cartesian coordinates. The unitary normalized gradient field is:

2 $$\nabla S=\frac{2(x-{x}_{0})+2(y-{y}_{0})+(z-{z}_{0})}{\sqrt{({(2(x-{x}_{0}))}^{2}+({(2(y-{y}_{0}))}^{2}+({(2(z-{z}_{0}))}^{2}}}$$

The norm of the vectors of ▽*S* can be weighted as function of the distance to the center of the sphere:

3 $${Q}_{a}={(-\mathrm{1)}}^{a}\frac{\nabla S}{|\nabla S{|}^{p}}$$

where $p\in {\rm{I}}R$ controls the decay of the vectors’ magnitude and *a* ∈ {0, 1} makes the field centripetal or centrifuge, respectively. Then, *W* = *Q*_0_ + *Q*_1_ is a vector field that moves from *Q*_0_ to *Q*_1_. In our approach, *Q*_0_ and *Q*_1_ correspond to the user introduced points and ${\mathcal M}$  = |*W*| is a 3D feature map indicating how valuable each voxel is for the segmentation. In terms of magnitude, ${\mathcal M}$ has high intensity in the region between the centers of *Q*_0_ and *Q*_1_ and low vector magnitude elsewhere, indicating to the network that the region between the two points has high interest for the segmentation. Changing *p* affects the strength of the interaction between the two points, as shown in Fig. 4. Namely, a lower *p* increases the focus on the central region but also increases its overall volume, whereas a high *p* leads to more spherical regions of interest surrounding the points. Note that if no points exist, then ${\mathcal M}$ is a zero-value tensor with the same size of the input volume.

### iW-Net for nodule segmentation

The proposed nodule segmentation scheme is adaptation of the 3D U-Net^13^. As shown in Fig. 5, iW-Net is composed of two auto-encoders: the first outputs an initial segmentation, which is then used as an input for the second block to produce the corrected segmentation. Each of the auto-encoders has a reduced number of filters in the encoding and decoding parts in comparison to the 3D U-Net, resulting in less parameters to tune and thus easing the back-propagation process.

We include the proposed segmentation weight map ${\mathcal M}$ by concatenating it to the initial feature maps of the encoding part of the second block of the model since preliminary experiments showed a significant performance drop if ${\mathcal M}$ was included on the upsampling part only. In fact, adding ${\mathcal M}$ on the initial part of segmentation correction block ensures that all weights of the model are affected by these external features. Due to the skip connections, ${\mathcal M}$ is also included on the final segmentation layer, thus directly affecting the model’s output.

### Loss function

iW-Net predicts a 3D map of the probability of each voxel belonging to the nodule. We use a two-term loss function, where the first is based on the Intersection over Union (IoU):

4 $${ {\mathcal L} }_{{\rm{IoU}}}=1-{\rm{IoU}}=1-\frac{\sum \,{I}_{t}\circ {I}_{p}}{\sum \,({I}_{t}+{I}_{p})-\sum \,{I}_{t}\circ {I}_{p}},$$

where *I*_*t*_ and *I*_*p*_ are the ground truth mask and the soft prediction mask, respectively, and ○ is the Hadamard product. The second term aims at forcing the network to have into account the manually introduced points by evaluating if there are segmentation points in the defined region of interest:

5 $${ {\mathcal L} }_{{\rm{attraction}}}=1-\frac{\sum \,(( {\mathcal M}  > \gamma )\circ {I}_{p})}{\sum \,( {\mathcal M}  > \gamma )},$$

where *γ* ∈ [01] controls the extent of the region of interest. The global loss ${\mathcal L}$ is the linear combination of Eqs 4 and 5:

6 $$ {\mathcal L} ={\lambda }_{1}{ {\mathcal L} }_{{\rm{IoU}}}+\mathrm{(1}-{\lambda }_{1}){ {\mathcal L} }_{{\rm{attraction}}}$$

where *λ*_1_ controls the relative importance of the terms.

### Dataset and training details

iW-Net was developed using the LIDC-IDRI^6^ dataset, which contains 1 012 LDCT scans with variable slice thickness. In this dataset, nodules with diameter ≥3 *mm* have voxel-wise annotations from up to 4 different expert radiologists and the corresponding inter-observer agreement level is indicative of how likely an abnormality is in fact a nodule. The dataset also contains a numeric description $\in {\mathbb{N}}$ of several nodule characteristics. Namely, nodule texture ∈ [1, 5] indicates the opacity of the nodule, with 1 being a pure non-solid nodule and 5 a pure solid nodule. We considered the 888 scans used for the LUNA16 challenge^18^ and studied 2 284 nodules (some samples were discarded due to annotation inconsistencies, poor scan reconstruction or excessive slice thickness). From those, 1 593, 1 190 and 790 have agreement level ≥2, ≥3 and ≥4, respectively. In our experiments, a nodule is considered non-solid if it has an average texture ≤2, solid if = 5 and sub-solid otherwise. For an agreement level ≥2, the dataset has 135 non-solid, 300 sub-solid and 1 695 solid nodules.

All nodules were collected by patching a 51 × 51 × 51 *mm* cube centered at the average center of mass of the specialists annotations and were then isotropically resized to 64 × 64 × 64 voxels. The intensity of the volume image was linearly mapped from [−1000 400] Hounsfield Units to [0 1]. Adam^19^ was used as optimizer (learning rate 0.001) and the network was trained using a batch size of 8 samples.

The dataset was artificially augmented by performing random rotations, translations, flips and zooms. For each epoch, user input was simulated by selecting the two most distant points on the middle axial slice of the segmentation. All agreement levels were considered to account for the inter-observer variability and thus no segmentation combination was performed, *i.e*. the same nodule was paired with different viable ground-truths to train the model. Furthermore, iW-Net was evaluated via stratified 5-fold cross-validation with partition at scan level and we used 20% of the training for validation. All hyper-parameters were found via random search^20^ with 100 search steps. At each step, $\{{\lambda }_{1},\gamma ,p\} \sim U\mathrm{([0,}\,\mathrm{1])}$, where *U* is an uniform distribution. Optimization was performed on the validation set of the first train-test split.

iW-Net was trained in two steps. The first block was initially trained separately using ${ {\mathcal L} }_{{\rm{IoU}}}$ until the validation loss stopped improving for 3 epochs. The weights were then frozen and the entire iW-Net was trained using ${\mathcal L}$, the output of the first segmentation block and the artificially generated user interaction until the loss stopped improving for 5 epochs. Since each nodule can have multiple segmentations (one per expert), iW-Net had to perform different corrections according to the expert’s annotation and the respective simulated user input. Experiments were performed on an Intel Core i7-5960X, 32 Gb RAM, 2× GTX1080 desktop with Python 3.5 and Keras 2.2. Code is available at https://github.com/gmaresta/iW-Net.

### Experiments and evaluation

iW-Net produces pixel-wise predictions ∈[0 1], which are thresholded at 0.5 for the model’s evaluation. The predictions are evaluated in terms of 3D Intersection over Union (IoU) and Average Surface Distance (ASD), as follows:

7 $${\rm{IoU}}(S,\hat{S})=\frac{(S\cap \hat{S})}{(S\cup \hat{S})}$$

8 $${\rm{ASD}}(S,\hat{S})=\frac{1}{2}(\frac{1}{{N}_{S}}\mathop{\sum }\limits_{i}^{{N}_{S}}\,{\rm{\min }}(d({S}_{i},\hat{S}))+\frac{1}{{N}_{\hat{S}}}\mathop{\sum }\limits_{i}^{{N}_{\hat{S}}}\,{\rm{\min }}(d({\hat{S}}_{i},S)))$$

where *S* is the expert’s annotation, $\hat{S}$ is the model’s prediction, *N*_*S*_ and ${N}_{\hat{S}}$ are the number of surface elements, *d* is the Euclidean distance (mm) and min is the minimum operation.

For each nodule, the average inter-observer IoU performance is computed by iteratively considering one expert’s annotation as the ground-truth and the remaining as predictions and then averaging the results. For instance, the inter-observer IoU performance in an agreement level 4 nodule is the average of 12 = 4 annotators × 3 predictions IoU results. For better comparison with the observers, iW-Net is only evaluated in nodules with agreement level ≥2. The segmentation performance is also analyzed in terms of nodule radius and texture. We consider the radius of each nodule as the average of the equivalent spherical radius of all the annotators.

#### Experiment 1

We study the performance of the non-guided segmentation unit (the first block of iW-Net) using as comparison the average inter-observer agreement and the segmentation produced using the 3D U-Net^13^. This U-Net is trained and tested on the aforementioned dataset. Due to computational constraints, the batch size is reduced to 2. Evaluation is performed according to Eq. 9:

9 $$\overline{{\rm{IoU}}}=\frac{1}{N}\mathop{\sum }\limits_{n\mathrm{=1}}^{N}\,{\rm{IoU}}({S}_{n,j},{\hat{S}}_{j})$$

where *N* is the expert’s agreement level for nodule *j*, *i.e*. the number of radiologists that annotated that nodule. Since a nodule can have multiple segmentations, it is not expected that the model outperforms the inter-observer agreement.

#### Experiment 2

The goal of this experiment is to evaluate the impact of the user’s input on the segmentation of iW-Net. For that, we artificially generate user inputs on the axial plane of the slice that contains the nodule’s centroid. Similarly to the training procedure, the two most points distant points in the ground-truth boundary of that slice are selected.

The performance of the full iW-Net is compared with the output of the first block in terms of IoU and ASD for different nodule sizes and textures. As in a real case scenario, we consider that the experts can keep either the initial or the corrected (*Cr*) segmentation, according to which better fits their needs. The evaluation is thus performed via Eq. 10:

10 $$\overline{{\rm{Cr}}\,{\rm{IoU}}}=\frac{1}{N}\mathop{\sum }\limits_{n\mathrm{=1}}^{N}\,{\rm{\max }}\,({\rm{IoU}}({S}_{n,j},{\rm{Cr}}{\hat{S}}_{n,j}),{\rm{IoU}}\,({S}_{n,j},{\hat{S}}_{j}))$$

This principle is also applied to the ASD metric having as decision criteria the IoU, *i.e*., the same nodules are considered.

## Conclusion

We propose iW-Net, a novel lung nodule interactive segmentation scheme. Drawing a stroke of the nodule’s diameter and respective end-point extraction allows to generate a weight map ${\mathcal M}$, which is then used for altering the prediction of the network. Specifically, ${\mathcal M}$ is designed having into account the expected spherical shape of the nodules and the distance between the introduced points. To promote the influence of ${\mathcal M}$ in the resulting segmentation, this map is incorporated as a feature of the model and as a component of the loss function.

iW-Net allows to improve the segmentation of more than 75% of the studied nodules. In fact, in comparison to the baseline, our model (with user interaction) significantly improves the segmentation of nodules with radii [1, 4](*mm*), which are essential for referral. Likewise, using iW-Net improves the segmentation performance of nodules with all types of textures, specially the challenging non-solid nodules. Given the inherent subjectivity of lung nodule segmentation, iW-Net may be an important tool to add to CAD systems, removing the need for manual segmentation while providing an easy and fast method to correct the produced output if needed.

## Data Availability

This study was performed using the publicly available LIDC-IDRI dataset (https://wiki.cancerimagingarchive.net/display/Public/LIDC-IDRI). The source code is available at https://github.com/gmaresta/iW-Net.
